# Optimal First-Line Treatment for* Helicobacter pylori* Infection: Recent Strategies

**DOI:** 10.1155/2016/9086581

**Published:** 2016-12-13

**Authors:** Ju Yup Lee, Kyung Sik Park

**Affiliations:** Department of Internal Medicine, Keimyung University School of Medicine, Daegu, Republic of Korea

## Abstract

A new treatment strategy is needed, as the efficacy of triple therapy containing clarithromycin—the current standard treatment for* Helicobacter pylori* infection—is declining. Increasing antibiotic resistance of* H. pylori *is the most significant factor contributing to eradication failure. Thus, selecting the most appropriate regimen depending on resistance is optimal, but identifying resistance to specific antibiotics is clinically challenging. In a region suspected to have high clarithromycin resistance, bismuth quadruple therapy and so-called nonbismuth quadruple therapies (sequential, concomitant, and sequential-concomitant hybrid) are some first-line regimen options. However, more research is needed regarding appropriate second-line treatments after first-line treatment failure. Tailored therapy, which is based on antibiotic sensitivity testing, would be optimal but has several limitations for clinical use, and an alternative technique is required. A novel potassium-competitive acid blocker-based eradication regimen could be a valuable eradication option in the near future.

## 1. Introduction

Although triple therapy containing proton pump inhibitors (PPIs), amoxicillin, and clarithromycin has been recommended worldwide as the standard treatment for* Helicobacter pylori* infection, the need for a new treatment strategy has developed, since the efficacy of triple therapy is declining in most countries [1]. Several factors play a role in the failure of* H. pylori* eradication, but resistance to antibiotics is considered the major cause [2]. Hence, the present study examines the recommended first-line treatments in different countries and analyzes the problems associated with such regimens in association with antibiotic resistance, in an attempt to identify appropriate treatment strategies.

## 2. First-Line Treatment Recommendations and the Problem of Antibiotic Resistance

The 2013 revision of the Korea Guideline for* H. pylori* recommends triple therapy containing clarithromycin as the first-line treatment and bismuth quadruple therapy in regions with suspected clarithromycin resistance [3]. However, according to a recent meta-analysis conducted in Korea, the eradication rate with triple therapy containing clarithromycin has been in significant decline for the last 10 years [4] as a result of antibiotic resistance of* H. pylori. *In fact, worldwide reports and a Korean report of primary antibiotic resistance in* H. pylori* suggest that the resistance rate is >20% for clarithromycin, >40% for metronidazole, and >10% for quinolones [5].

Similar to Korea, China recommends a 7–14-day regimen of the standard triple therapy containing clarithromycin and amoxicillin as first-line treatment [6], but the eradication rate is known to be <80% [7]. Hence, bismuth quadruple therapy is recommended as first-line treatment in regions where* H. pylori* resistance to clarithromycin exceeds 15–20% [6]. Because there are considerable regional differences in* H. pylori* antibiotic resistance in China, regional characteristics should be considered when determining treatment [8, 9]; most regions have high metronidazole and clarithromycin resistance rates. For example, the* H. pylori* rate of resistance to metronidazole in Shanghai is approximately 60–70%, while the rate of resistance to clarithromycin is approximately 20–38% [10].

On the other hand, Japan uses lower doses of antibiotics for eradication than Korea or China [11]. The recommended primary treatment comprises twice-a-day administration of a standard dose of PPI, amoxicillin 750 mg, and clarithromycin 200 or 400 mg for 7 days, while the secondary treatment comprises twice-a-day administrations of PPI, amoxicillin 750 mg, and metronidazole 250 mg for 7 days. Fourteen-day treatment or bismuth quadruple treatment is not recommended as first- or second-line therapy in Japan [7]. The eradication rate associated with first-line treatment in Japan is 70%, while that for second-line treatment is 90%, maintaining an eradication rate >95% with these two regimens [12, 13]. One notable phenomenon in Japan is that it has relatively low antibiotic resistance rates of* H. pylori *compared to those of other countries; thus, the high eradication rates in Japan are a testament to the fact that triple therapy containing clarithromycin or metronidazole remains efficacious in regions with low antibiotic resistance rates. However, the prevalence of* H. pylori* antibiotic resistance is growing in Japan as well. According to a multi-institutional survey in Japan, the clarithromycin resistance rate of* H. pylori* was 18.9% in 2002, which escalated to 27.2% in 2006 [14]. Although there are some regional differences, metronidazole resistance largely remains low at 2.1–24.0% [14].

The 2012 revision of the European guideline for* Helicobacter* introduces a treatment strategy for clarithromycin-resistant* H. pylori* [15]. Whereas triple therapy containing clarithromycin is recommended as first-line treatment in regions with a clarithromycin resistance rate <20%, bismuth quadruple therapy, sequential therapy, or concomitant therapy is recommended as first-line treatment in regions with clarithromycin resistance >20% [15]. According to prior reports, despite the fact that sequential or concomitant therapies contain clarithromycin, they overcome clarithromycin resistance to a certain extent, with approximately 75–95% therapeutic success rates.

## 3. First-Line Treatment Options for Cases with High Antibiotic Resistance

**Figure 1:**
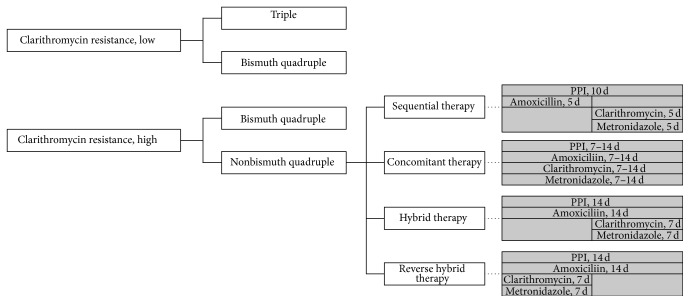
The recent treatment strategy and administration method for nonbismuth quadruple therapy. Dose of each drug: proton pump inhibitor (PPI) standard dose twice a day, amoxicillin 1.0 g twice a day, clarithromycin 500 mg twice a day, and metronidazole 500 mg twice a day.

Alternatives to primary treatment with clarithromycin triple therapy include bismuth quadruple therapy and so-called nonbismuth quadruple therapies: sequential, concomitant, and sequential-concomitant hybrid. The treatment strategy and administration method for each regimen are shown in Figure 1 [15, 16].

### 3.1. Bismuth Quadruple Therapy

Traditional bismuth quadruple therapy involves 7- or 14-day administration of a standard dose of PPI (twice daily), bismuth 120 mg (4 times/day), tetracycline 500 mg (4 times/day), and metronidazole 500 mg (3 times/day). Theoretically, bismuth easily reaches* H. pylori* since it is released into the gastric mucosa and is associated with low resistance; thus, there have been arguments that quadruple therapy containing bismuth should preferentially be employed as the first-line treatment for* H. pylori *infection instead of triple therapy containing a PPI [17]. Discussions on whether bismuth quadruple therapy can replace PPI triple therapy as first-line treatment are ongoing, but for now, this is considered the most appropriate alternative, especially in regions with high clarithromycin resistance. In a meta-analysis conducted in 2003, there was no statistically significant difference between PPI triple therapy and bismuth quadruple therapy [18], and a meta-analysis conducted in 2013 of 12 randomized controlled trials (RCTs) reported that the eradication rate associated with bismuth quadruple therapy was 77.6%, while that associated with PPI triple therapy was 68.9% (relative risk [RR], 1.13; 95% confidence interval [CI], 1.07–1.18) [19]. However, interpretations of meta-analyses should be performed with caution since different studies established different selection and exclusion criteria, used different types of PPIs, and used different doses for quadruple therapy. One new consideration for bismuth quadruple therapy is the development of three-in-one capsules comprising bismuth, metronidazole, and tetracycline [20, 21]. The intention-to-treat (ITT) eradication rate associated with quadruple therapy using three-in-one capsules is 80% (95% CI, 73.9–84.9%) in Europe [20] and 87.7% (95% CI, 82.2–93.2%) in the United States [21], showing relatively satisfactory performance. However, this regimen consisted of 14 dosing units (3 three-in-one capsules 4 times a day, with a PPI twice a day) daily for 10–14 days [20]; therefore, compliance is a concern when these many doses are prescribed in actual clinical practice. Antibiotic resistance is also a factor contributing to bismuth quadruple therapy failure. Bismuth quadruple therapy contains tetracycline and metronidazole, but tetracycline resistance rates are reportedly low worldwide [5, 22, 23], implying that metronidazole resistance would influence the therapeutic outcomes of bismuth quadruple therapy.

### 3.2. Sequential Therapy

Zullo et al. [24] first introduced sequential therapy in 2000, reporting a 98% ITT eradication rate (95% CI, 94.3–100%). According to three meta-analyses based on RCTs conducted in Italy before 2008, an ITT analysis revealed that the eradication rate with sequential therapy was 91.0–93.5%, which was significantly higher than that with triple therapy (75.7–79.2%) [25–27]. ITT analyses revealed that the eradication rates with sequential treatment in RCTs conducted in Europe (Italy, Spain) after 2008 were generally >80%, and although this was a slight decline compared to earlier implementation periods, the eradication rates with sequential treatment remain higher than those with triple therapies [28–34]. However, ITT eradication rates in Asia, including Korea, were approximately 10% lower than that of Italy in the earlier days of implementation [35, 36], which is presumed to be due to the regional characteristics of* H. pylori* antibiotic resistance in Asia. Although the dual resistance rate to clarithromycin and metronidazole in Italy is only 3.5–4.3% [37, 38], the rates in Asian regions are higher (9.6–44.7%) [39, 40]. Another cause is the use of metronidazole instead of tinidazole, as suggested in the original regimen proposed in Italy [41]. This, at least in part, could explain why sequential therapy achieved inferior success rates outside Italy, where metronidazole has generally been used. Many studies have reported that the eradication rate associated with sequential therapy slightly decreases in the presence of monoresistance to clarithromycin but remains markedly higher than that of standard triple therapy [42–45]. However, sequential therapy is associated with a higher eradication rate than that of standard triple therapy, but antibiotic resistance would reduce such results to below the expected rate. For instance, one meta-analysis reported eradication rates for sequential and standard therapy in the presence of clarithromycin resistance to be 70.3% and 33.8%, respectively [27]. The eradication rate with sequential therapy also decreases for monometronidazole resistance, but there has been little relevant research.

In addition, sequential therapy was proven to be highly effective and superior to triple therapies in children [46] and elderly patients [47]; however, no similar data were available for either concomitant or bismuth quadruple therapy.

The drugs administered in the first and last half are different in a sequential regimen; therefore, it is likely that patient compliance could decrease due to dosing complexity. However, the compliance with sequential therapy was almost the same as that with standard triple therapy (97.4% and 96.8%, resp.) in a previous meta-analysis [25]; furthermore, most studies showed high compliance with sequential therapy in clinical practice [48–50].

### 3.3. Concomitant Therapy

Concomitant therapy involves the simultaneous administration of PPI, amoxicillin, clarithromycin, and metronidazole. ITT analyses conducted in two meta-analyses on studies published up until the early 2000s revealed that the eradication rate with concomitant therapy was approximately 90%, exceeding that of standard triple therapy [51, 52]. Similarly, a meta-analysis of 15 studies (1,723 patients) up until 2011 showed that ITT eradication rates associated with concomitant therapy were approximately 90%, which was higher than that with standard triple therapy [53]. The average ITT eradication rate with concomitant therapy reported by a follow-up meta-analysis published in 2012 on 19 studies (2,070 patients) was 88% [54]. There were no differences in the side effects with concomitant therapy versus triple therapy, and none of the prior studies reported severe side effects associated with concomitant therapies [51]. Several studies reported that concomitant therapy has therapeutic effects similar to that of sequential therapy [31, 33, 55, 56] but is associated with more frequent side effects [31]. The compliance with concomitant therapy was similar to that for sequential therapy [45, 56, 57]. However, higher compliance was achieved with concomitant therapy than with sequential therapy in a meta-analysis of RCTs in Chinese regions [58]. Concomitant therapy is known to demonstrate better eradication rates in patients with clarithromycin- or metronidazole-resistant strains [59, 60], and the selection of secondary antibiotics after first-line concomitant therapy failure remains challenging.

### 3.4. Sequential-Concomitant Hybrid Therapy

Hybrid therapy is a combination of sequential and concomitant therapy and consists of administering PPI and amoxicillin for 14 days and adding clarithromycin and metronidazole in the last 7 days. A multi-institutional study in Taiwan that first described the use of hybrid therapy reported an ITT eradication rate of 97.4% and a per-protocol eradication rate of 99.1% [61], which was about 5% higher than the eradication rate with 14-day sequential therapy performed by the same institution [62]. However, in a study in Korea, there was no difference in the eradication rates with 14-day hybrid therapy and 14-day sequential therapy [63]; similarly, there was no difference in the eradication rates with 14-day hybrid therapy and 14-day concomitant therapy conducted in Italy and Spain [64]. A recent meta-analysis showed there was no significant difference in eradication rates between hybrid and sequential (RR 1.01, 95% CI: 0.92–1.11) or concomitant therapy (RR 0.98, 95% CI: 0.93–1.02) [16]. However, it is difficult to generalize a conclusion regarding hybrid therapy because the number of studies remains small, while studies that compared hybrid therapy with sequential and concomitant therapies involved different administration durations [57]. Nevertheless, eradication rates associated with hybrid therapy have been determined to exceed those of standard triple therapy. Recently, Hsu et al. [65] showed a high eradication rate with reverse hybrid therapy. Reverse hybrid therapy consists of a PPI and amoxicillin for 14 days, with addition of clarithromycin and metronidazole for the first 7 days. The ITT eradication rate of reverse hybrid therapy was 93.6%, which was superior to that of standard triple therapy [65]. The drug compliance rates for hybrid and reverse hybrid therapy were 96.2% [16] and 96.8% [65], respectively, and were comparable to those for standard triple therapy. Hybrid therapy displayed a slightly higher compliance rate than concomitant therapy (95.8% versus 93.2%) in a recent meta-analysis including 12 RCTs of hybrid therapy [57].

### 3.5. Tailored Therapy

Since antibiotic resistance is the main cause of eradication failure, a tailored therapy that selects the most appropriate regimen to overcome antibiotic resistance would be the optimal treatment. However, tailored therapies are complex and costly. Therefore, a new method to detect antibiotic resistance is needed. One such solution is the polymerase chain reaction (PCR) kit, which uses the dual-priming oligonucleotide-based multiplex PCR test [66]. This kit identifies the presence of point mutations of clarithromycin 23S rRNA that are known to be associated with resistance, namely, A2142G and A2143G, using a PCR [67]. The test can be performed with a sample of gastric mucosa and takes only a few hours, making it relatively simple to use; its sensitivity and specificity are approximately 80–85% [68].

### 3.6. Potassium-Competitive Acid Blocker

The potassium-competitive acid blocker (P-CAB), vonoprazan, could improve eradication rates by increasing the intragastric pH and thus increasing bacterial antibiotic susceptibility. Vonoprazan 20 mg demonstrated a more rapid and sustained acid-inhibitory effect than esomeprazole 20 mg or rabeprazole 10 mg [69]. Recent studies revealed that P-CAB based triple therapy was more effective than PPI-based triple therapy as a first-line* H. pylori* eradication method [70–73]. Furthermore, even in the presence of clarithromycin-resistant strains, P-CAB-based triple therapy showed good eradication rates that were superior to those for PPI-based triple therapy (76.1% versus 40.2%) [74].

## 4. Conclusion

Triple therapy containing clarithromycin is now largely considered a “legacy therapy” because its therapeutic efficacy decreases even with a clarithromycin resistance of 7–10% [75]; thus, it has become ineffective in regions with high clarithromycin resistance. Hence, sequential or concomitant regimens are recommended as first-line treatment in regions with high clarithromycin resistance rates. However, the therapeutic effects of these two regimens in the presence of dual resistance to clarithromycin and metronidazole have yet to be determined [60]. In such cases, the hybrid regimen could be an alternative [64]. The bismuth quadruple regimen could be a good first-line alternative regardless of clarithromycin resistance rates, but its efficacy parallels that of triple therapy and it requires a 14-day administration, which would undermine medication compliance. When first-line therapy of the bismuth quadruple or nonbismuth quadruple regimen fails, an appropriate second-line regimen should contain a quinolone; however, there is little research to support this, and it should be noted that many regions have high quinolone resistance rates. Tailored therapy based on antibiotic sensitivity testing would be the optimal approach but has several limitations for clinical use, calling for the development of a new alternative technique. A novel P-CAB-based regimen would be a valuable* H. pylori* eradication option.
